# New Flexon-based reagents for tissue-specific Auxin-Inducible Degradation and for characterizing Cre and Flp drivers in *C. elegans*

**DOI:** 10.17912/micropub.biology.001315

**Published:** 2024-08-19

**Authors:** Julia Wittes, Iva Greenwald

**Affiliations:** 1 Dept. of Biological Sciences, Columbia University, New York, New York, USA

## Abstract

A Flexon stop cassette interrupts translation of a coding region until it is excised by a recombinase to allow for gene expression. We have expanded options for Auxin-Inducible Degradation by generating Flexon-based transgenes for tissue-specific expression of the ubiquitin ligase substrate recognition component TIR1 or the variant TIR1(F79G) after excision of the Flexon by Cre recombinase. We also describe Flexon-based tester transgenes to facilitate gathering accurate information about the expression pattern of Cre and Flp recombinase drivers that can be used in conjunction with any conditional expression reagents that utilize these recombinases.

**Figure 1. Flexon-based reagents for tissue-specific expression of TIR1 and for characterizing Cre and Flp recombinase excision patterns:**
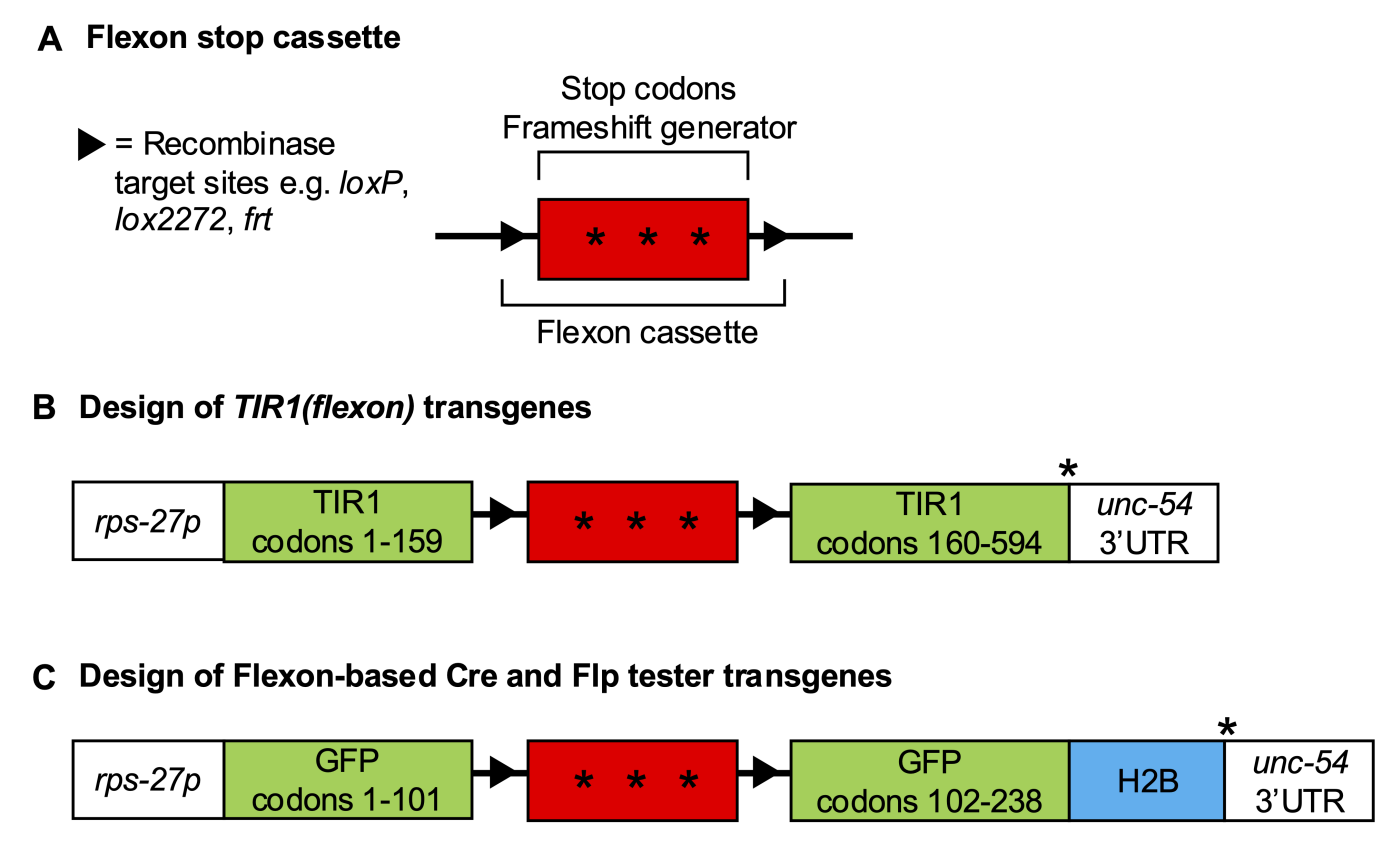
A) A Flexon stop cassette is an artificial exon that contains stop codons and a frameshift generator (***), leading to termination of protein translation and nonsense-mediated decay of the transcript. Triangles represent recombinase target sites and the thin line represents intronic sequences flanking the artificial exon. For further information and considerations about Flexon design and placement see Shaffer and Greenwald (2022). B) *TIR1(flexon)* is expressed under the control of a strong ubiquitous promoter from *rps-27* ( *rps-27p)* . In the absence of Cre, the Flexon cassette interrupts TIR1 translation after codon 159. In the presence of Cre, the Flexon is excised and the complete TIR1 protein is expressed under the control of *rps-27p* . *TIR1F79G(flexon)* has an identical design to *TIR1(flexon)* . C) The design of Cre and Flp testers was based on the design of *arTi361 [rps-27p::GFP(flexon)::H2B::unc-54 3'UTR]* (Shaffer and Greenwald 2022). Nuclearly-localized GFP is expressed under the control of *rps-27p* after excision in the presence of an appropriate Cre or Flp driver. H2B represents the sequence of a histone gene, *his-58* . All transgenes reported in this study use the neutral *unc-54* 3'UTR.

## Description

The Auxin-Inducible Degradation (AID) system is a valuable method for conditional protein depletion in *C. elegans* (Zhang et al., 2015) . In this system, a degron tag (also represented as "AID") is added to the protein of interest, making it a potential substrate for recognition by the *Arabidopsis* TIR1 protein; the addition of auxin promotes association of TIR1 with the AID moiety, leading to ubiquitination and proteasome-dependent degradation of the degron-tagged protein. Spatial control over protein depletion is provided by tissue-specific expression of TIR1 and temporal control can be provided by the addition of auxin. A modified AID system uses TIR1(F79G) (Uchida et al., 2018; Hills-Muckey et al., 2022) , and both systems are useful additions to the *C. elegans* genetic toolbox.

In addition to being specific, appropriate drivers for tissue-specific AID should also result in strong expression of TIR1 to maximize the effectiveness of target protein depletion in the presence of auxin. However, a tissue-specific promoter may not produce a sustained high level of gene expression. In such cases, a stop cassette such as a Flexon ( Figure 1A ), which interrupts gene expression until excised via flanking recombinase sites, can provide strong, sustained expression. For example, in our work on the somatic gonad, we have relied extensively on a promoter derived from *ckb-3* that is highly and specifically expressed in the somatic gonad precursors Z1 and Z4, but not in their descendants (Kroetz and Zarkower 2015; see also Shaffer and Greenwald 2022) . However, specific, strong, sustained expression in the somatic gonad has been achieved by combining a *ckb-3p::Cre* driver transgene with a transgene in which the strong promoter of a ribosomal protein gene drives expression of fluorescent proteins (Shaffer and Greenwald 2022) or biologically active proteins (O'Keeffe and Greenwald 2022) after excision of a Flexon in the somatic gonad. We adapted this approach to create Flexon-based reagents for strong, tissue-specific expression of TIR1 by generating Flexon-interrupted transgenes that express TIR1 under the control of *rps-27p* ( Figure 1B ). A high level of TIR1 expression is thus ensured in tissues where Cre recombinase is expressed.

In many existing TIR1 transgenes, the level and tissue-specificity of expression of TIR1 has been evaluated based on a fluorescent protein fused to TIR1 (e.g. Zhang et al., 2015) or co-expressed using a 2A peptide (e.g. Xiao et al., 2023). A limitation of this general approach is that it precludes the ability to use a fluorescent marker of the same color. Our Flexon-based TIR1 transgenes are not fluorescently tagged, so any fluorescent markers may be used.

It is important to characterize the excision pattern of recombinase drivers when using any reagents that depend on them for excision, including the *TIR1(flexon)* reagents described here. Promoters that appear tissue-specific based on expression of fluorescent proteins have been observed to produce broader excision patterns when used to express recombinases because of transient historical expression or low-level expression not readily detected using fluorescent reporter proteins (Ruijtenberg and van den Heuvel 2015; Tenen and Greenwald 2019) . To facilitate characterization of recombinase drivers, we developed Flexon-based reporters for Cre or Flp recombinase such that nuclearly-localized GFP is produced after excision of the Flexon ( Figure 1C ). Cre recombinase excision patterns can be visualized by generating strains containing the Cre driver and a miniMos transgene such as *arTi452* or *arTi361* (see Methods). In addition, we also developed a strain to facilitate the generation and characterization of new Cre drivers in one step: plasmids for Cre expression can be directly injected into the strain GS10037 , which contains *arSi159* , a single-copy insertion generated by MosSCI at *ttTi5605 II* . This strain is convenient for direct injection because Cre drivers cloned in miniMos vectors that have G418 or hygromycin B selection markers (e.g. pCFJ910 or pCFJ1662) can be easily selected in this background. Finally, we also generated a Flexon-based reporter containing *frt* sites ( *arTi461 )* , which can be used to characterize Flp drivers. Using this transgene we were able to confirm that the Flexon system works with the Flp-FRT system, as was previously proposed (Shaffer and Greenwald 2022) .

## Methods

All constructs were cloned using Gibson Assembly (NEB HiFi reagent). All miniMos transgenes were generated in the N2 strain background by standard methods (Frøkjær-Jensen et al., 2014). The transgene *arSi159* was made using mosSCI: by injecting into EG6699 [ *ttTi5605 ; unc-119 ( ed3 ); oxEx1578 [eft-3p::GFP + Cbr-unc-119 ]* ] (Frøkjær-Jensen et al., 2008).

Table 1 summarizes information about the new transgenes generated in this study, and Table 2 provides primer sequences that can be used for genotyping.

**TIR1 Flexon-based expression reagents.** pJSW49 *[rps-27p::TIR1(flexon)::unc-54 3'UTR]* was cloned in the pCFJ1662 miniMos vector, which includes a hygromycin B selection cassette (Frøkjær-Jensen et al., 2014). The TIR1 sequence is derived from pLZ31 (Zhang et al., 2015) and was modified by replacing the first synthetic intron (after codon 159) with a Flexon sequence identical to the one reported in Shaffer and Greenwald (2022). The sequence encoding the F-Box domain and first leucine-rich repeat (LRR) of TIR1 precede the Flexon and the sequence encoding the final 4 LRR domains and the remainder of TIR1 follow the Flexon, which guards against the production of a functional protein in the absence of Cre expression. This plasmid was injected to make the transgene *arTi362* via standard miniMos protocols.

pJSW87 *[rps-27p::TIR1F79G(flexon)::unc-54 3'UTR]* was derived from pJSW49 by introducing the F79G mutation, changing the codon TTC to GGA (Uchida et al., 2018; Hills-Muckey et al., 2022) , by Gibson assembly. This plasmid was used to make the miniMos transgene *arTi443* .

**New Flexon-based recombinase testers.** The previously-described tester *arTi361 [rps-27p::GFP(flexon)::H2B::unc-54 3'UTR]* has a Flexon flanked by *lox2272* sites, and was derived from the plasmid pHK001 (Shaffer and Greenwald 2022) . pHK001 was modified to create the new plasmids described here.

**Cre recombinase testers.** *arTi452* was derived from pJSW89, in which the *lox2272* sites of pHK001 were replaced with *loxP* . *arSi159* was derived from pJSW102, which has the same insert as pJSW89, but it was generated in the pCFJ151 mosSCI backbone (Frøkjær-Jensen et al., 2008). The availability of equivalent testers with different *lox* sites allows for maximum flexibility for their use as cell markers when combined with other genome engineered loci that have *lox* scars or paired *lox* sites.

**Flp recombinase tester.** *arTi461* was derived from the plasmid pJSW96, in which the *lox2272* sites of pHK001 were replaced by *frt* sites.

**Table:** 

**Transgene**	**Plasmid**	**Genotype**	**Target**	**Plasmid backbone, selection**	**Genetic map location**
*arTi362*	pJSW49	*rps-27p::TIR1(flexon)::unc-54 3'UTR*	*lox2272*	pCFJ1662, Hygromycin B	-19.98 V
*arTi443*	pJSW87	*rps-27p::TIR1F79G(flexon)::unc-54 3'UTR*	*lox2272*	pCFJ1662, Hygromycin B	+21.99 V
arTi452 *	pJSW89	*rps-27p::GFP(flexon)::H2B::unc-54 3'UTR*	*loxP*	pCFJ910, G418	-4.81 I
arTi461 *	pJSW96	*rps-27p::GFP(flexon)::H2B::unc-54 3'UTR*	*frt*	pCFJ910, G418	-3.80 I
*arSi159*	pJSW102	*rps-27p::GFP(flexon)::H2B::unc-54 3'UTR*	*loxP*	pCFJ151, *Cbr-unc-119 (+) *	*ttTi5605* II

**Table 1.**

The plasmids listed in Table 1 will be deposited at Addgene or available upon request. The following strains will be deposited at the CGC or available upon request.

GS9820 *arTi443*

GS9402 *arTi362*

GS9847 *arTi452*

GS9922 *arTi461*

GS10037 *arSi159 ; unc-119 ( ed3 )*

* GS9407 *arTi361 [rps-27p::GFP(flexon)::H2B::unc-54 3'UTR]* is equivalent to *arTi452* and *arTi461* except with *lox2272* sites (Shaffer and Greenwald 2022) .

**Table:** 

**Transgene**	**Genotyping primers**	**Band sizes**
*arTi362*	oJSW172 = GGGATACAGTGTCAAGGCTAGTG oJSW183 = CACGTCCTTGATGATTCTCGGCA oCF1590 = CGATAAATATTTACGTTTGCGAGAC	No transgene: 674 bp band Transgene: 306 bp band
*arTi443*	oJSW330 = AACCGAGAGAGACGTAGACAC oJSW331 = GATTTGTCAGCCATTCGTCTG oCF1591 = AAAAATGGCTCGATGAATGG	No transgene: 481 bp band Transgene: 251 bp band

**Table 2.**
